# The sigma-1 receptor agonist fluvoxamine alleviates endotoxin-induced acute lung injury in mice

**DOI:** 10.3389/fphar.2026.1818198

**Published:** 2026-04-28

**Authors:** Emese Ritter, Kata Csekő, Ádám Hosszú, Ákos R. Tóth, Dóra Hargitai, László Kereskai, Andrea Fekete, Zsuzsanna Helyes

**Affiliations:** 1 Department of Pharmacology and Pharmacotherapy, University of Pécs, Medical School, Pécs, Hungary; 2 National Laboratory for Drug Research and Development, Budapest, Hungary; 3 MTA-SE Lendület “Momentum” Diabetes Research Group, Budapest, Hungary; 4 Pediatric Center, Semmelweis University, Budapest, Hungary; 5 Department of Pathology and Experimental Cancer Research, Semmelweis University, Faculty of Medicine, Budapest, Hungary; 6 Department of Pathology, University of Pécs, Medical School, Pécs, Hungary; 7 HUN-REN-PTE Chronic Pain Research Group, Pécs, Hungary; 8 PharmInVivo Hungary Ltd., Pécs, Hungary

**Keywords:** acute lung injury, dexamethansone, fluvoxamine, lipopolysaccharide, sigma 1 receptor

## Abstract

**Introduction:**

Acute lung inflammation has recently gained increasing attention due to the high acute respiratory distress syndrome complications with subsequent fibrosis during the COVID-19 pandemic. Our group identified that the antifibrotic effect of the antidepressant fluvoxamine (FLU) in various organs is meditated via sigma-1 receptor (S1R) agonism. Since the actions of FLU on the inflammatory components have not been elucidated, this study investigated its effects in a mouse model of interstitial pneumonitis.

**Methods:**

Pneumonitis was induced in wild-type (WT) and S1R knockout (*S1r* ^*−/−*^) mice by intratracheal administration of lipopolysaccharide: 0.5 mg/kg LPS. Treatment groups were randomized into 1) phosphate-buffered saline (PBS) +vehicle, 2) LPS + vehicle, 3) LPS + FLU (*i.p.* 20 mg x bwkg^-1^) or 4) LPS + dexamethasone (*i.p.* 5 mg x bwkg^-1^) groups.

**Results:**

LPS reduced tidal volume, minute ventilation, peak expiratory, inspiratory and mid-tidal expiratory flows. Similarly to the reference compound dexamethasone FLU counteracted all effects in WT, but not in *S1r* ^*−/−*^ mice. Furthermore, FLU alleviated LPS-induced macrophage infiltration in both genotypes, but had no effect on lung edema or neutrophil accumulation. FLU downregulated inflammatory cytokines IL-1, IL-6, TNF-α, and MCP-1 in WT mice*,* similarly to dexamethasone, but not in *S1r* ^*−/−*^ mice.

**Conclusion:**

Overall, FLU mitigates LPS-induced pulmonary inflammation and functional deterioration primarily via S1R signaling, highlighting a receptor-specific mechanism underlying its protective effects. Thus, targeting S1R may be an effective and safe alternative to other therapeutic approaches, including glucocorticoids to treat inflammatory lung injury.

## Graphical Abstract

**Figure:**
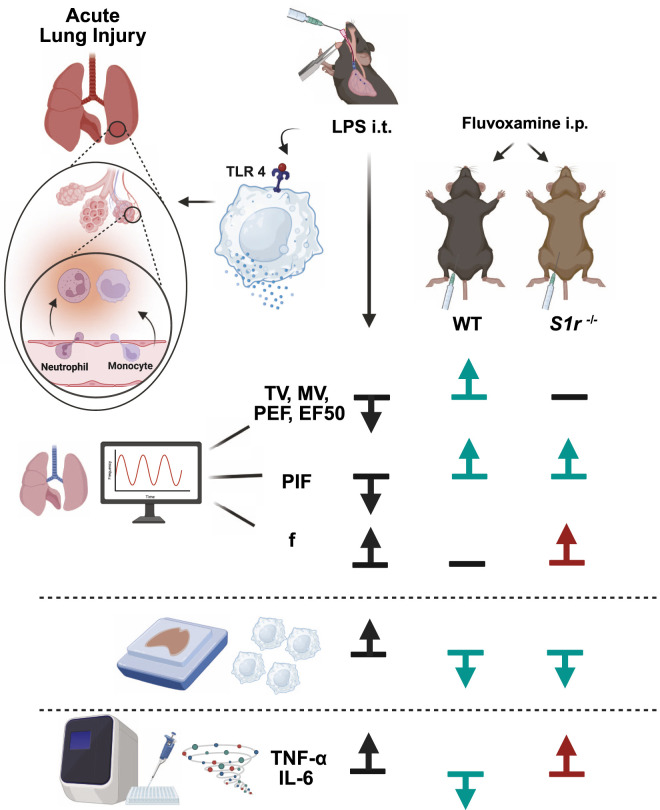
Created in BioRender. Bencze, N. (2026) https://BioRender.com/fs4l9po.

## 1 Introduction

Acute lung injury (ALI) and its more severe manifestation, acute respiratory distress syndrome (ARDS), are inflammatory conditions with up to a 50% mortality rate (Matthay et al., 2019). They are characterized by massive inflammatory cell infiltration and increased vascular permeability leading to edema, hypoxemia and ultimately respiratory failure (Ma et al., 2025).

The emergence of novel pathogens, such as SARS-CoV-2 in the COVID-19 pandemic, and therapy unresponsiveness have brought increasing global attention to ALI/ARDS, as reflected by a sharp surge in related publications since 2020.

Glucocorticoids are the current standard of care for ARDS, however, their therapeutic efficacy differs, with many patients showing limited response, or even higher mortality rate in certain patient subgroups besides the numerous adverse effects, such as secondary infections, hyperglycemia and myopathy (Li et al., 2024; Zhao et al., 2024). This inconsistency may be partly due to inappropriate timing or dosing, but also to the complex pathophysiological mechanisms of the partially explored inflammatory cascade (Chernov et al., 2026) To address this, numerous drug repurposing trials have been initiated to improve survival. Despite these efforts, many early treatment strategies have been retracted, and effective options for glucocorticoid-resistant ARDS are still lacking (Zhao et al., 2024). Thus, it is crucial to explore the key processes and molecular pathways, as well as to identify novel therapeutic targets and candidates for better treatment options.

The sigma-1 receptor (S1R) is a ligand-regulated chaperone protein that is ubiquitously expressed in various tissues (Aishwarya et al., 2021). Several studies have demonstrated the therapeutic potential of S1R agonists in central nervous system disorders, partially via their anti-inflammatory effects (Sałaciak and Pytka, 2022). In contrast, the role of S1R in peripheral organ pathologies has not been extensively investigated. In recent years, our research group has demonstrated the protective role of the S1R in experimental models of acute kidney injury and renal transplantation. These effects were closely linked to a marked suppression of pro-inflammatory cytokine expression and immune cell infiltration, highlighting S1R as a potent anti-inflammatory target (Hosszu et al., 2023; Toth et al., 2025). Additionally, we have shown that S1R activation mitigates endotoxin (lipopolysaccharide: LPS)-induced inflammation *in vitro* in renal proximal tubular epithelial cells (Balogh et al., 2024). Given the central role of inflammation in the pathogenesis of ALI and ARDS, it is highly likely that S1R activation confers similar protective benefits in the lung, making it a compelling therapeutic target.

A subset of ligands targeting S1R were identified during the extensive efforts to find novel tools to reduce SARS-CoV-2 infectivity (Gordon et al., 2020). Several antidepressants traditionally classified as selective serotonin reuptake inhibitors (SSRIs) were reported to exert additional S1R agonistic activity (Mas et al., 2022). Fluvoxamine (FLU) is a widely used and generally well-tolerated SSRI with high affinity for S1R, that emerged as the most promising anti-inflammatory candidate (Hashimoto et al., 2022; Narita et al., 1996; Ishikawa et al., 2007).

Intratracheal LPS-induced acute interstitial pneumonitis is a reproducible and widely used rodent model with a well-defined mechanism initiated by Toll-like receptor 4 (TLR4) activation on alveolar macrophages and consequent neutrophil influx (Domscheit et al., 2020). This leads to the release of proinflammatory cytokines along with reactive oxygen species and chemotactic factors, subsequently resulting in excessive inflammation, edema, and hemorrhage (Matute-Bello et al., 2008). This model has recently been characterized, optimized, and validated in mice by our group (Ritter et al., 2025). Although interstitial pneumonitis is induced by a bacterial endotoxin in this model, it is highly relevant to mimic the TLR4-triggered cytokine storm characteristic of ARDS with different etiologies including SARS-CoV-2 (Asaba et al., 2024).

Building on our previous findings, the present study aimed to investigate the effects of FLU and its mechanism via S1R activation using gene-deficient mice in the LPS-induced acute pneumonitis model with an integrative methodological approach. The obtained results may support S1R activation as a potential novel therapeutic strategy for inflammatory lung diseases.

## 2 Materials and methods

### 2.1 Animals

Experiments were performed on 8–10-week-old female C57BL/6J (Charles River Laboratories, Calco, Italy) and *S1r* ^*−/−*^ mice. *S1r* ^*−/−*^ *mice were* kindly gifted from Dr. Adrian Wong (Ottawa Hospital Research Institute, Ottawa, Canada). The mice used in the experiments were produced from heterozygous breeding pairs and assigned randomly to each experiment. The *S1r* ^*−/−*^ genotype was confirmed by PCR. Altogether 51 C57BL/6J and 61 *S1r* ^*−/−*^ mice were used in the study. All experiments were conducted in accordance with the ARRIVE guidelines and the 40/2013 (II.14) Government Decree on Animal Protection, and Consideration Decree of Scientific Procedures of Animal Experiments (XXVIII of 1998, 243/1988) and the European legislation (Directive 2010/63/EU). License was given by the Ethics Committee on Animal Research of University of Pécs, Pécs, Hungary, according to the Ethical Codex of Animal Experiments (licence No.: BA/73/00607-6/2021). Animals were kept in the minimal disease (MD) Laboratory Animal House of the Department of Pharmacology and Pharmacotherapy. Mice kept in the MD facility are non-pathogen free; their microbiological status is monitored regularly by GVG Diagnostics GmbH, Leipzig, Germany, based on FELASA recommendations. standard laboratory chow (SAFE® A03 pellets, SAFE®, Augy, France) and tap water *ad libitum*. Animals were housed in autoclaved open-type TII cages (367 × 140 × 207 mm) (Acéllabor Ltd., Vecsés, Hungary) on spruce wood fibrillated fibers (SAFE® 3-4 S, SAFE®, Augy, France) enriched by GLP fun tunnels (Innovo Ltd., Isaszeg, Hungary). LPS-treated animals were housed in a separate room of the Animal House. Standardized conditions were provided at constant temperature (20 °C–22 °C), 40%–55% humidity, and a 12-h light-dark cycle switched on at 6 a.m. and off at 6 p.m. Animal welfare was assessed by the mouse grimace scale throughout the study.

### 2.2 Experimental design

**FIGURE 1:**
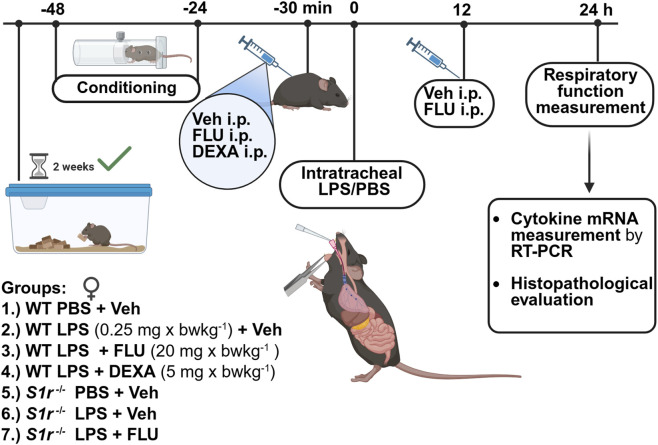
Experimental design. (PBS: phosphate-buffered saline, LPS: lipopolysaccharide, Vehicle (physiological saline solution), FLU: fluvoxamine, DEXA: dexamethasone, wild-type (WT), sigma-1 receptor knockout (S1r^−/−^)). Created in BioRender. Bencze, N. (2026) https://BioRender.com/kiu94y4.

Pulmonary inflammation was evoked by intratracheal (*i.t.*) LPS (L2630; *Escherichia coli* O111:B4; Merck KGaA, Darmstadt, Germany; 0.25 mg/kg dissolved in 60 mL phosphate-buffered saline (PBS)). WT and *S1r* ^*−/−*^ animals were randomized into 4 and 3 groups, respectively i) negative control mice received PBS *i.t.* and vehicle (Veh, physiological saline solution) intraperitoneally (*i.p.*) 30 min prior and 12 h after PBS administration (PBS + Veh), ii) *i.t.* LPS-treatment with *i.p.* Veh 30 min prior and 12 h after induction (LPS + Veh), iii) *i.t.* LPS-treatment with fluvoxamine maleate (FLU) (Sigma-Aldrich (St. Luis, MO, United States) *i.p.* 20 mg x bwkg^-1^ 30 min prior and 12 h after induction) (LPS + FLU). To pharmacologically validate the model WT LPS-administered animals were randomized into an additional positive control group with the gold standard reference compound dexamethasone (DEXA) (Dexa-ratiopharm 4 mg/mL; Teva Pharmaceuticals Ltd., Debrecen, Hungary; *i.p.* 5 mg x bwkg^−1^) (LPS + DEXA). Since dexamethasone has a long duration of action it was only administered 30 min before LPS. To minimize injection-related bias (stress, pain) LPS + DEXA group was also treated with *i.p.* Veh 12 h after induction. After 24 h, respiratory function measurement was performed, then animals were anesthetized with ketamine-xylazine (*i.p.* 100 mg/kg and 5 mg/kg, respectively). Lung tissues were harvested, measured, and frozen for qPCR measurement, or formalin-fixed for histopathology (Figure 1).

### 2.3 Respiratory function measurement

Respiratory functions were measured in awake animals using Buxco FinePointe Non-Invasive Airway Mechanics (NAM) restrained plethysmography (DSI Harvard Bioscience Inc., St. Paul, MN, United States) 24 h after LPS/PBS administration. Respiratory function parameters (breathing frequency, tidal volume, minute ventilation, peak expiratory and inspiratory flow, tidal-mid expiratory flow, inspiratory and expiratory time) were recorded every 2 s for 5 min. To minimize stress-related bias induced by restraint, conditioning to immobilization 24 and 48 h before induction, as well as a 15-min acclimatization period preceded baseline measurement (Ritter et al., 2025).

### 2.4 Histopathological assessment

Lungs were fixed in 6% paraformaldehyde and embedded in paraffin. The number of neutrophil granulocytes was assessed on 5 µm hematoxylin-eosin-stained sections. Ten lung septa were selected randomly from each section, and the number of neutrophils was counted in a blinded manner by a pathologist. CD68-immunopositive cells were counted in ten randomly selected parenchymal areas with 20′ magnification (Olympus BX51, Tokio, Japan). Inflammatory cells were expressed as total number/10^6^ mm^2^ tissue using ImageJ software (version 1.54 g). Tissue samples were deparaffinized, rehydrated and incubated in EnVision Flex Target Retrieval Solution (pH 9) (Agilent, Santa Clara, CA US). Endogenous peroxidase activity was quenched by 3% hydrogen peroxide. The sections were washed and incubated in blocking solution, then treated with a 1:1000 dilution of rabbit polyclonal anti-CD68 (ab125212; Abcam, Cambridge, United KIngdom) antibody. Slides were incubated with anti-rabbit secondary antibody conjugated with HRP (Histols®-R) and visualized by Histols®-DAB (Histopathology Ltd, Pécs, Hungary) and the EnVision system.

### 2.5 Quantitative RT-PCR

Lung samples were homogenized in Precellys 24 Evolution homogenizer (Bertin, Montigny-le-Bretonneux, France). Total RNA was extracted using the RNeasy RNA Isolation Kit (Qiagen, Germantown, MD) according to the manufacturer’s instructions. RNA quality and quantity were measured by NanoDrop One Spectrophotometer (Thermo Scientific, Waltham, MA, United States). RNA samples were reverse transcribed using Maxima First Strand cDNA Synthesis Kit for RT-qPCR (Thermo Fisher Scientific, Waltham, MA, United States). RT-qPCR was performed on the LightCycler 96 system (Roche Diagnostics, Mannheim, Germany), SYBR Green I Master enzyme mix (Roche Diagnostics, Mannheim, Germany), and 10 pmol μL^−1^ of each specific primers (IDT, Coralville, Iowa, United States) (Table 1). Results were analyzed by LightCycler 96 software version 1.1.0.1320 (Roche Diagnostics, Mannheim, Germany). All data were normalized using the housekeeping gene *Rn18S*.

**TABLE 1:** Primer sequences for real-time quantitative PCR. *bp*–base pair.

Gene name	Regular name	Primer sequence	Product length (bp)	Annealing temperature (°C)
*Rn18s*	Mouse ribosomal 18S ribosomal RNA	Forward: 5′ACT​TAA​AGG​AAT​TGA​CGG​AAG​GG 3′	183	60
		Reverse: 5′GAA​TTA​ACC​AGA​CAA​ATC​GCT​CC 3′		
*Il1b*	Mouse IL- 1β	Forward: 5′TGC​CAC​CTT​TTG​ACA​GTG​AT 3′	180	60
		Reverse: 5′CCA​CAG​CCA​CAA​TGA​GTG​AT 3′		
*Tnf*	Mouse TNF-α	Forward: 5′AGA​CCC​TCA​CAC​TCA​GAT​CA 3′	196	60
		Reverse: 5′ACC​TGG​GAG​TAG​ACA​AGG​TAC 3′		
*Il1a*	Mouse IL-1α	Forward: 5′TCC​TTC​TAT​GAT​GCA​AGC​TAT​GG 3′	244	57
		Reverse: 5′ATC​TGG​GTT​GGA​TGG​TCT​CTT 3′		
*IL6*	Mouse IL-6	Forward: 5′CAG​ACC​TGT​CTA​TAC​CAC​TTC​AC 3′	93	57
		Reverse: 5′TTG​CCA​TTG​CAC​AAC​TCT​TTT​C 3′		
*Ccl2*	Mouse MCP-1	Forward: 5′CCC​AAA​GAA​GCT​GTA​GTT​TTT​GTC 3′	76	57
		Reverse: 5′TAA​TGT​ATG​TCT​GGA​CCC​ATT​CC 3′		

### 2.6 Statistical analysis

Statistical evaluation was performed by effect size analysis and GraphPad Prism v8 (GraphPad Software, San Diego, CA, United States) software. Data with high effect size, indicated by Hedges’g >0.8, were marked on the figures. Supplementary Tables S1–S4 show the results of effect size analysis, one-way ANOVA followed by Dunnett’s multiple comparisons test and two-way ANOVA followed by Tukey’s *post hoc* test with significance p < 0.05.

## 3 Results

### 3.1 FLU rescues respiratory function in LPS-induced ALI

**FIGURE 2:**
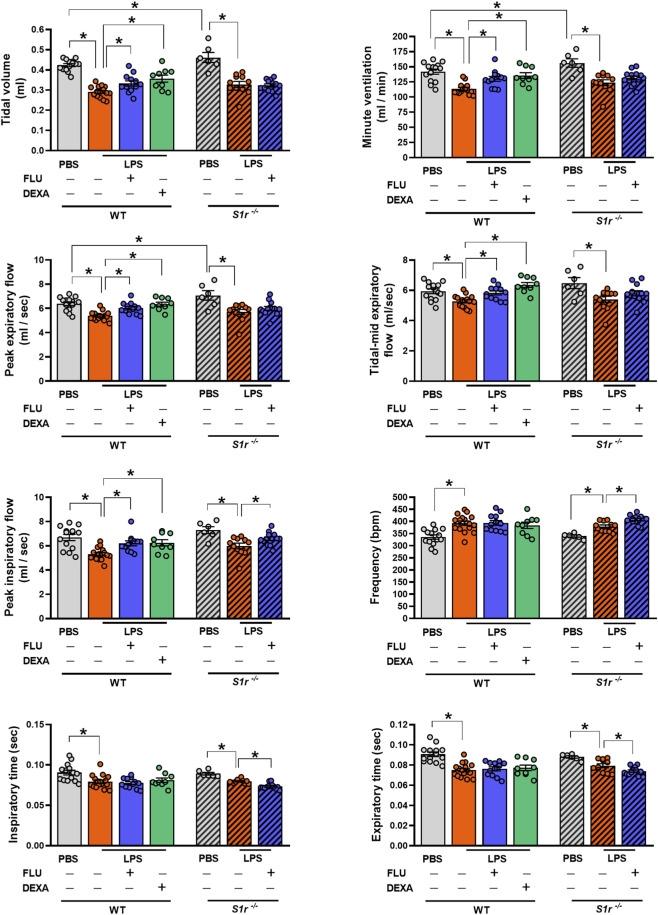
Respiratory function measured by restrained plethysmography in wild-type (WT) and sigma-1 receptor knockout (S1r^−/−^) mice. (PBS: phosphate-buffered saline, LPS: lipopolysaccharide, vehicle (physiological saline solution), FLU: fluvoxamine, DEXA: dexamethasone). Data are expressed as means ± SEM, n = 6-16/group (Effect size analysis, *Hedges’g >0.8 vs. corresponding group).

Intratracheal LPS instillation induced a significant decrease in tidal volume, minute ventilation, peak expiratory-, mid-tidal expiratory- and peak inspiratory flow, as well as inspiratory and expiratory time, whereas an increase in breathing frequency (Figure 2; Supplementary Tables S1, S4). These alterations were observed in both WT and *S1r* ^*−/−*^ mice. FLU counteracted the LPS-induced tidal volume, minute ventilation, peak expiratory, tidal mid-expiratory, and peak inspiratory flow decrement in the WT mice, similarly to the gold standard reference compound DEXA. However, it did not affect breathing frequency, inspiratory and expiratory time. Moreover, FLU further aggravated these latter parameters in the gene-deficient mice. Altogether, except for peak inspiratory flow, FLU showed no or aggravating impact in the *S1r* ^*−/−*^ mice, suggesting that the beneficial effects are mainly S1R mediated.

### 3.2 FLU ameliorates LPS-induced macrophage, but does not affect neutrophil granulocyte infiltration and edema formation

**FIGURE 3:**
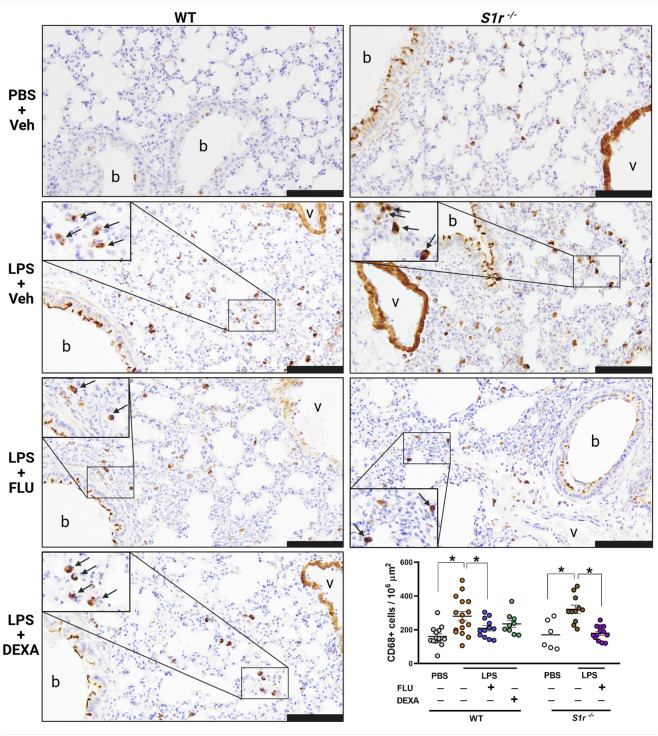
Representative histopathological images and quantification of inflammatory CD68^+^ macrophage numbers. Lung sections were obtained from PBS-administered control, LPS-evoked vehicle (Veh)-, fluvoxamine (FLU)-, or dexamethasone (DEXA)-treated wild-type (WT) and S1r knockout (S1r^−/−^) mice. Quantitative results are expressed as means ± SEM with the individual data points, n = 6-16/group (Effect size analysis, *Hedges’g >0.8 vs. corresponding group; CD68 immunostaining; scale bar: 100 μm; b: bronchiole, v: vessel, arrow: CD68^+^ macrophages).

**FIGURE 4:**
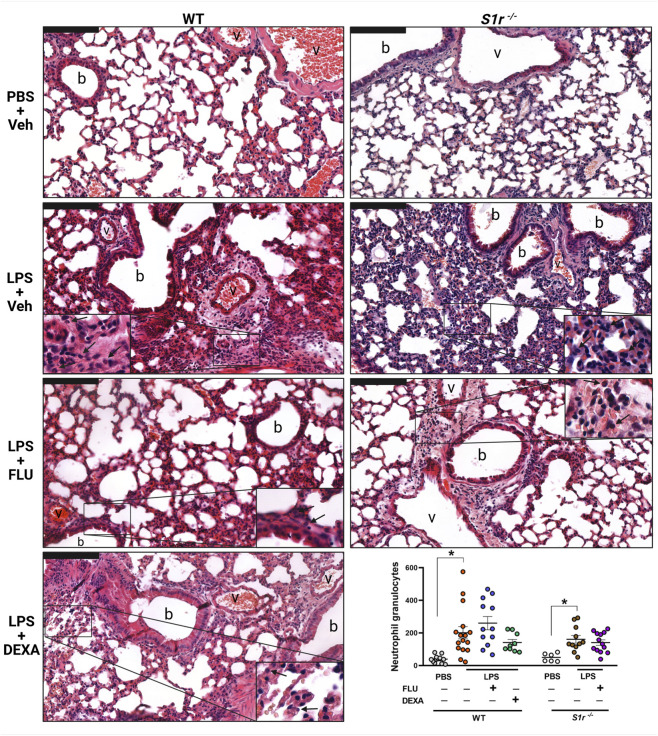
Representative histopathological images and quantification of neutrophil granulocyte infiltration. Lung sections were collected from PBS-administered control, LPS-administered vehicle (Veh)-, fluvoxamine (FLU)-, or dexamethasone (DEXA)-treated wild-type (WT) and sigma-1 receptor knockout (*S1r* ^*−/−*^ *)* mice. Quantitative results are expressed as means ± SEM with individual data points, n = 6-16/group (Effect size analysis, *Hedges’g > 0.8 vs. corresponding group; hematoxylin-eosin (HE) staining; scale bar: 100 μm; b: bronchiole, v: vessel, arrow: neutrophil granulocyte).

LPS induced substantial CD68^+^ macrophage infiltration in the lung tissue, in all groups that was diminished by FLU in both wild-type and S1r^−/−^ animals (Figure 3; Supplementary Tables S1, S4). However, FLU did not influence neutrophil granulocyte count in either group (Figure 4; Supplementary Tables S1, S4). In contrast to FLU, the anti-inflammatory reference compound DEXA had no effect on inflammatory cell infiltration.

### 3.3 LPS decreased body weight and induced pulmonary edema in WT and S1r^−/−^ mice

**FIGURE 5:**
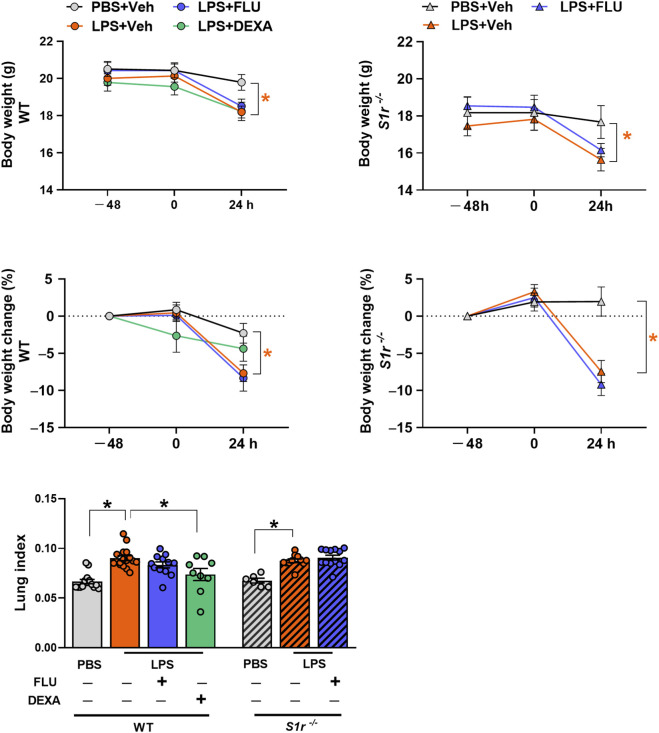
Body weight, body weight change and lung index. (PBS: phosphate-buffered saline, LPS: lipopolysaccharide, Vehicle (physiological saline solution), FLU: fluvoxamine, DEXA: dexamethasone, wild-type (WT), sigma-1 receptor knockout (S1r^−/−^)). Data are expressed as means ± SEM, n = 6-16/group (Effect size analysis, *Hedges’g >0.8 vs. corresponding group).

LPS evoked a decrease in body weight and body weight change and an increase in lung index (total lung weight/10 g body weight) indicative of the development of pulmonary edema 24 h after induction in WT and *S1r* ^*−/−*^ mice. These alterations were not influenced by FLU treatment in either genotype. On the contrary, dexamethasone significantly reduced lung edema formation (Figure 5; Supplementary Tables S1, S2, S4).

### 3.4 FLU alleviates inflammatory cytokine expression in WT, but not in S1r^−/−^ mice

**FIGURE 6:**
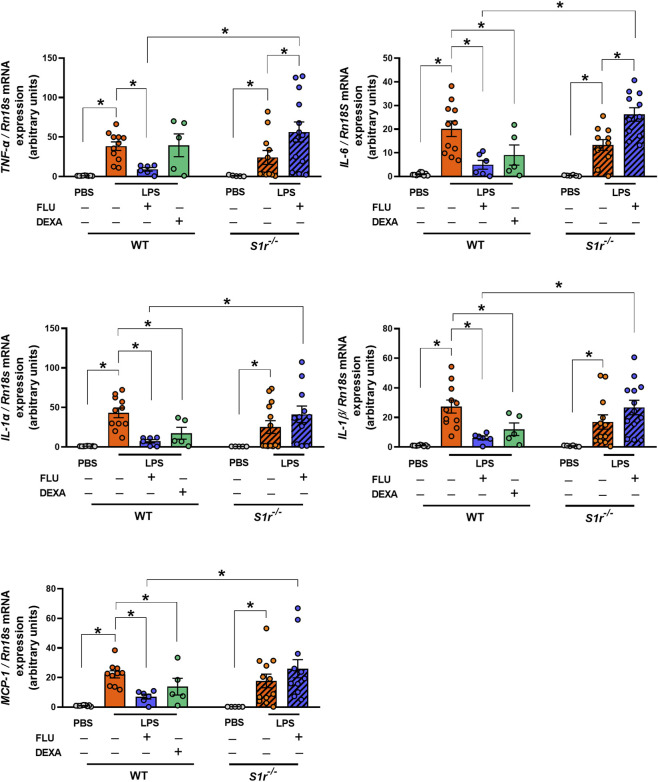
Inflammatory cytokine expressions (TNF-α, IL-6, IL-1α, IL- 1β, MCP1) measured by quantitative RT-PCR. (PBS: phosphate-buffered saline, LPS: lipopolysaccharide, Vehicle (physiological saline solution), FLU: fluvoxamine, DEXA: dexamethasone, wild-type (WT), sigma-1 receptor knockout (S1r^−/−^)). Data are expressed as means ± SEM*,* n = 5-13/group (Effect size analysis, *Hedges’g >0.8 vs. corresponding group).

LPS induced a robust increase in the levels of TNF-α, IL-6, IL-1α, IL-1β, and MCP-1 inflammatory cytokines in both genotypes. FLU diminished the LPS-induced alterations in the WT but not in the *S1r* ^*−/−*^ mice; moreover, it aggravated the upregulated levels of IL-6 and TNF-α in the gene-deficient animals (Figure 6; Supplementary Tables S3, S4). DEXA, used as a reference compound, downregulated all cytokines except TNF-α; in contrast, FLU showed broader cytokine suppression, suggesting a potentially more effective anti-inflammatory profile.

## 4 Discussion

In this study, we provide the first evidence that the S1R agonist, FLU mitigates respiratory dysfunction and inflammatory responses in a pharmacologically validated model of LPS-induced acute interstitial pneumonitis. FLU markedly attenuated LPS-evoked impairments in respiratory function and reduced inflammatory cytokine expression in WT mice. These effects were abolished in S1r^−/−^ animals, which provides strong *in vivo* support for an S1R–mediated mechanism.

The COVID-19 pandemic, associated with a high mortality rate due to ARDS, has challenged healthcare systems worldwide, emphasizing the importance of identifying novel drug targets and drug repurposing for pharmacological interventions (Chakraborty et al., 2021; Rodrigues et al., 2022). During the second wave of the COVID-19 pandemic, a comprehensive proteomic analysis was conducted to elucidate a protein-protein interaction map between SARS-CoV-2 viral proteins and the human host proteome, revealing more than 60 drug targets and candidate compounds for repurposing. The identification of S1R as a critical host protein for SARS-CoV-2 replication has provided a rationale for modulating S1R for the development of novel antiviral drugs (Gordon et al., 2020; Vela, 2020). Furthermore, our group has patented the therapeutic application of S1R agonists for the treatment of fibrosis in multiple organs, including the lung (PCT/HU2015/000014), and has demonstrated the anti-inflammatory effects of S1R activation in experimental models of both acute and chronic kidney injury (international patent: PCT/HU2017/050051) (Hosszu et al., 2023; Balogh et al., 2024).

S1R is a non-opioid intracellular chaperone protein localized in the mitochondria-associated endoplasmic reticulum (ER) membrane (Hayashi and Su, 2007). Through its chaperone function, it plays a key role in vital cellular functions primarily by regulating calcium flow, protein folding, and modulating ER stress sensor pathways (Hayashi, 2019). S1R is abundantly expressed in the central nervous system, but it has been found in many peripheral tissues as well, including the lungs (Couly et al., 2022).

In aged mice, the absence of S1R was associated with significant alveolar structural alterations including increased inflammatory infiltration and upregulated expression of surfactant proteins and nuclear factor kappa b (NF-kb), a central mediator of inflammatory responses. Moreover, elevated levels of profibrotic markers were observed in S1R gene-deficient mice, underlining the role of *S1r* in remodeling as well (Remex et al., 2023). Along with these recent findings suggesting a protective role of S1R in ageing-related lung damage, our results showed that even in young mice, S1R deficiency alters some crucial respiratory parameters, such as an increased tidal volume, minute ventilation, and peak expiratory flow. An elevated tidal volume is typically associated with elevated minute ventilation as well as an increase in expiratory flow. Mice with lower body weight could exhibit higher relative metabolic rates that could explain the baseline differences. However, a study investigating the basal metabolic rates of *S1r* ^*−/−*^ mice demonstrated no substantial differences in metabolism and energy expenditure despite the lower body weight and fat mass in knockout animals (Li et al., 2022). Another plausible explanation could be the relatively larger lung volume in smaller animals; however, the lung index of control mice exhibited no differences. Taken together, these statistically significant but biologically less relevant differences are not conclusive regarding the role of S1R in regulating basal respiratory function, as no other airway parameter exhibited basal differences between the WT and knockout genotypes. The LPS-evoked functional alterations, such as tidal volume, minute ventilation, peak expiratory, tidal mid-expiratory, and peak inspiratory flow, were counteracted by FLU in the WT mice. However, FLU treatment either had no beneficial effect or even worsened respiratory parameters in *S1r* ^*−/−*^ mice except for peak inspiratory flow.

Our results also demonstrate that FLU alleviates LPS-induced pneumonitis via reducing TNF-α, IL-6, IL-1α, IL- 1β, MCP1 expression in the WT but not in the *S1r* ^*−/−*^ mice. These data are supported by a study indicating that S1R plays a key role in suppressing inflammatory cytokine production in the LPS-induced septic shock mouse model (Rosen et al., 2019). Similarly, intraperitoneal LPS-induced increases in serum IL-6 levels were mitigated by FLU in WT mice but aggravated in *S1r* ^−/−^ mice (Rosen et al., 2019). In the ovalbumin-induced allergic asthma mouse model, S1R activation by a selective agonist PRE-084 also reduced inflammatory cell infiltration, cytokine production (IL-4, IL-5, IL-13), and consequent remodeling (Jiang et al., 2022). In the context of potential anti-SARS-CoV-2 therapy, it has been hypothesized that FLU may modulate the inositol-requiring enzyme 1 alpha (IRE1a) upstream stress sensor pathways through S1R agonism, therefore downregulating inflammatory ER stress (Rosen et al., 2019; Asadi Anar et al., 2022).

Interestingly, FLU reduced macrophage infiltration independently of S1R activation, indicating the involvement of distinct molecular pathways. FLU alleviated LPS-induced upregulation of intercellular adhesion molecule 1 (ICAM-1), vascular cell adhesion molecule-1 (VCAM-1) in an endothelial cell culture (Rafiee et al., 2016). Since the initial steps of inflammation including recruitment and migration of immune cells are mediated by cell adhesion molecules (Singh et al., 2023), FLU might inhibit CD68-positive cell accumulation via this mechanism. Despite alleviating cellular inflammatory components, FLU did not substantially decrease edema formation, suggesting its minor effect on venular plasma protein extravasation.

Although there is no universal consensus regarding the optimal therapeutic regime of glucocorticoid treatment, it is considered among the first line of ARDS pharmacological management (Li et al., 2024). Therefore, we used deaxamethasone, as the gold standard reference compound to pharmacologically validate the LPS model in WT mice. The results show that DEXA effectively alleviated LPS-induced tidal volume, minute ventilation, peak expiratory flow, tidal-mid expiratory and peak inspiratory flow decrease, lung edema formation, as well as *IL-6*, *IL-1*α, *IL-1*β and *MCP-1* expression, however, it did not substantially influence breathing frequency, inspiratory and expiratory time, inflammatory cell infiltration and TNF-α expression. We have administered the long-acting DEXA once during the 24 h protocol, 30 min before the induction. However, a recent study of ARDS/diffuse alveolar damage in ICR mice found that optimal timing and duration of glucocorticoid treatment substantially affect functional and histological outcomes (Chernov et al., 2026). This could be the plausible explanation for the inconsistency of dexamethasone effect in our study.

A limitation of the study is that only female mice were used. However, based on literature data there is no substantial sex-related difference in the LPS-induced ALI model (Ritter et al., 2025). Although the potential S1R agonistic effect of 17β-estradiol cannot be excluded (Sałaciak and Pytka, 2022), based on our results LPS-evoked inflammatory changes were similar in both WT and S*1r* ^*−/−*^ mice, therefore it does not seem to considerably influence the final conclusion.

The efficacy of FLU as a repurposed drug or as an adjuvant therapy in initial COVID-19 treatment remains undecided. A recent meta-analysis of six randomized trials found no significant overall benefit of FLU in reducing clinical deterioration or hospitalization, however, two of the six studies reported a lower rate of clinical decline (Vatvani et al., 2023), a subsequent meta-analysis of randomized controlled trials, open-label studies, and retrospective studies suggested its protective effect on disease progression and mortality. Optimal outcomes are critically dependent on treatment timing and higher dosage in outpatients with early symptoms (Prasanth et al., 2024), but FLU is indeed a promising option for ARDS therapy.

Collectively, our preclinical results support S1R activation as a viable therapeutic strategy and suggest that S1R agonism may represent an effective and safe alternative to other therapeutic approaches, including glucocorticoids in inflammatory lung diseases.

## Data Availability

All relevant data is contained within the article. The original contributions presented in the study are included in the article/Supplementary Material, further inquiries can be directed to the corresponding author.
