# Supplementary material for: The sigma-1 receptor agonist fluvoxamine alleviates endotoxin-induced acute lung injury in mice
Source: Front Pharmacol. 2026 Apr 28;17:1818198. doi: 10.3389/fphar.2026.1818198 (PMC13161069; doi:10.3389/fphar.2026.1818198)
Supplement: Supplementary file 1 [file Supplementaryfile1.docx]

**Supplementary material**

**The sigma-1 receptor agonist fluvoxamine alleviates endotoxin-induced acute lung injury in mice**

Emese Ritter^*^, Kata Csekő^*^, Ádám Hosszú*, Ákos R. Tóth, Dóra Hargitai, László Kereskai, Andrea Fekete^*^ and Zsuzsanna Helyes^*^

*E.R, K.Cs and Á.H share the first authorship as well as A.F and Zs.H share the last authorship (equal contributions)

Corresponding author: Kata Csekő; email: [cseko.kata@pte.hu](mailto:cseko.kata@pte.hu); Tel: +36-72-538-212 (ext. 38230)

**Supplementary Table 1.** LPS-induced alterations in pulmonary functions (tidal volume, minute ventilation, peak expiratory, tidal-mid expiratory and peak inspiratory flow, frequency, inspiratory and expiratory time), in the numbers of CD68 immunopositive cells and neutrophil granulocytes and in the lung index in fluvoxamine- and dexamethasone-treated wild-type and *S1r* gene-deficient (*S1r^-/-^*) mice. Large effect size **(**Hedges’ g>0.8) was considered significant as well as p<0.05 analyzed by one-way ANOVA followed by Dunnett’s multiple comparisons test and marked as bold, n = 6-16 mice/group, *Hedges’g > 0.8 vs corresponding group.

|  | **Tidal volume** |  | **LPS** | | **PBS** | | **Minute**  **ventilation** |  | **LPS** | | **PBS** | |
| --- | --- | --- | --- | --- | --- | --- | --- | --- | --- | --- | --- | --- |
|  |  |  | **WT** | ***S1r*** ^-/-^ | **WT** | ***S1r*** ^-/-^ |  |  | **WT** | ***S1r*** ^-/-^ | **WT** | ***S1r*** ^-/-^ |
| **PBS** |  | **g** | **4.65***  **<0.0001** | **2.37***  **<0.0001** | − | − |  | **g** | **2.21***  **<0.0001** | **1.90***  **0.0003** | − | − |
|  |  | **p** |  |  |  |  |  | **p** |  |  |  |  |
| **FLU** |  | **g** | **1.13***  **0.0142** | 0.13  0.9400 | **2.40***  **<0.0001** | **3.26***  **<0.0001** |  | **g** | **1.43***  **0.0053** | 0.55  0.3305 | 0.76  0.0712 | **1.74***  **0.0032** |
|  |  | **p** |  |  |  |  |  | **p** |  |  |  |  |
| **DEXA** |  | **g** | **1.67***  **0.0003** | − | **1.66***  **0.0004** | − |  | **g** | **1.93***  **0.0007** | − | 0.41  0.5359 | − |
|  |  | **p** |  |  |  |  |  | **p** |  |  |  |  |
|  | **Peak expiratory flow** |  | **LPS** | | **PBS** | | **Tidal-mid expiratory flow** |  | **LPS** | | **PBS** | |
|  |  |  | **WT** | ***S1r*** ^-/-^ | **WT** | ***S1r*** ^-/-^ |  |  | **WT** | ***S1r*** ^-/-^ | **WT** | ***S1r*** ^-/-^ |
| **PBS** |  | **g** | **2.11***  **<0.0001** | **1.62***  **0.0014** | − | − |  | **g** | **2.27***  **0.0014** | **0.95***  **0.0087** | − | − |
|  |  | **p** |  |  |  |  |  | **p** |  |  |  |  |
| **FLU** |  | **g** | **1.51***  **0.0025** | 0.45  0.5021 | 0.67  0.1403 | **1.43***  **0.0093** |  | **g** | **1.26***  **0.0128** | 0.45  0.0985 | **1.06***  0.8840 | 0.66 |
|  |  | **p** |  |  |  |  |  | **p** |  |  |  |  |
| **DEXA** |  | **g** | **2.28***  **<0.0001** | − | 0.06  0.9963 | − |  | **g** | **2.42***  **<0.0001** | − | 0.06  0.1296 | − |
|  |  | **p** |  |  |  |  |  | **p** |  |  |  |  |
|  | **Peak inspiratory flow** |  | **LPS** | | **PBS** | | **Frequency** |  | **LPS** | | **PBS** | |
|  |  |  | **WT** | ***S1r*** ^-/-^ | **WT** | ***S1r*** ^-/-^ |  |  | **WT** | ***S1r*** ^-/-^ | **WT** | ***S1r*** ^-/-^ |
| **PBS** |  | **g** | **1.81***  **<0.0001** | **1.65***  **0.0011** | − | − |  | **g** | **1.61***  **0.0002** | **2.08***  **0.0004** | − | − |
|  |  | **p** |  |  |  |  |  | **p** |  |  |  |  |
| **FLU** |  | **g** | **1.46***  **0.0072** | **0.89***  0.0670 | 0.55  0.2527 | **1.16***  0.0652 |  | **g** | 0.03  0.9997 | **1.46***  **0.0010** | **1.67***  **0.0004** | **4.07***  **<0.0001** |
|  |  | **p** |  |  |  |  |  | **p** |  |  |  |  |
| **DEXA** |  | **g** | **1.48***  **0.0105** | − | 0.48  0.3847 | − |  | **g** | 0.24  0.8826 | − | **1.31***  **0.0071** | − |
|  |  | **p** |  |  |  |  |  | **p** |  |  |  |  |
|  | **Inspiratory time** |  | **LPS** | | **PBS** | | **Expiratory time** |  | **LPS** | | **PBS** | |
|  |  |  | **WT** | ***S1r*** ^-/-^ | **WT** | ***S1r*** ^-/-^ |  |  | **WT** | ***S1r*** ^-/-^ | **WT** | ***S1r*** ^-/-^ |
| **PBS** |  | **g** | **1.14***  **0.0028** | **2.95***  **<0.0001** | − | − |  | **g** | **2.04***  **<0.0001** | **1.52***  **0.0028** | − | − |
|  |  | **p** |  |  |  |  |  | **p** |  |  |  |  |
| **FLU** |  | **g** | 0.24  0.9029 | **1.94***  **0.0002** | **1.40***  **0.0011** | **4.18***  **<0.0001** |  | **g** | 0.18  0.9481 | **0.99***  **0.0236** | **1.82***  **<0.0001** | **3.59***  **<0.0001** |
|  |  | **p** |  |  |  |  |  | **p** |  |  |  |  |
| **DEXA** |  | **g** | 0.19  0.9455 | − | **0.92***  **0.0363** | − |  | **g** | 0.29  0.8281 | − | **1.58***  **0.0004** | − |
|  |  | **p** |  |  |  |  |  | **p** |  |  |  |  |
|  | **CD 68+ cells/ 10^6^ μm^2^** |  | **LPS** | | **PBS** | | **Neutrophil granulocytes** |  | **LPS** | | **PBS** | |
|  |  |  | **WT** | ***S1r*** ^-/-^ | **WT** | ***S1r*** ^-/-^ |  |  | **WT** | ***S1r*** ^-/-^ | **WT** | ***S1r*** ^-/-^ |
| **PBS** |  | **g** | **1.30***  **0.0004** | **1.75***  **0.0003** | − | − |  | **g** | **1.42***  **0.0008** | **1.60***  **0.0035** | − | − |
|  |  | **p** |  |  |  |  |  | **p** |  |  |  |  |
| **FLU** |  | **g** | **0.80***  0.0594 | **2.19***  **<0.0001** | **0.82***  0.2892 | 0.15  0.9369 |  | **g** | 0.39  0.3999 | 0.28  0.6678 | **2.20***  **<0.0001** | **1.64***  **0.0131** |
|  |  | **p** |  |  |  |  |  | **p** |  |  |  |  |
| **DEXA** |  | **g** | 0.45  0.4007 | − | **1.19***  0.0814 | − |  | **g** | 0.46  0.4497 | − | **2.53***  0.0999 | − |
|  |  | **p** |  |  |  |  |  | **p** |  |  |  |  |
|  | **Lung index** |  | **LPS** | | **PBS** | |  |  |  |  |  |  |
|  |  |  | **WT** | ***S1r*** ^-/-^ | **WT** | ***S1r*** ^-/-^ |  |  |  |  |  |  |
| **PBS** |  | **g** | **2.47***  **<0.0001** | **3.10***  **<0.0001** | − | − |  |  |  |  |  |  |
|  |  | **p** |  |  |  |  |  |  |  |  |  |  |
| **FLU** |  | **g** | 0.64  0.3062 | 0.40  0.4901 | **1.75***  **0.0016** | **2.76***  **<0.0001** |  |  |  |  |  |  |
|  |  | **p** |  |  |  |  |  |  |  |  |  |  |
| **DEXA** |  | **g** | **1.18***  **0.0037** | − | 0.53  0.3495 | − |  |  |  |  |  |  |
|  |  | **p** |  |  |  |  |  |  |  |  |  |  |

**Supplementary Table 2.** LPS-induced alterations in body weight and body weight change in response to fluvoxamine and dexamethasone treatment in wild-type and *S1r^-/-^* mice. Large effect size (Hedges’ g>0.8) was considered significant, and p<0.05 analyzed by two-way ANOVA followed by Dunnett’s multiple comparisons test was further considered statistically significant and marked as bold, n = 5-16 mice/group, *Hedges’g > 0.8 vs corresponding group.

|  | **Body weight** |  | **LPS** | | **PBS** | | **Body weight change** |  | **LPS** | | **PBS** | |
| --- | --- | --- | --- | --- | --- | --- | --- | --- | --- | --- | --- | --- |
|  |  |  | **WT** | ***S1r*** ^-/-^ | **WT** | ***S1r*** ^-/-^ |  |  | **WT** | ***S1r*** ^-/-^ | **WT** | ***S1r*** ^-/-^ |
| **PBS** |  | **g** | **1.09***  **0.0157** | **0.93***  0.1414 | − | − |  | **g** | **1.11***  **0.0127** | **1.81***  **0.0053** | − | − |
|  |  | **p** |  |  |  |  |  | **p** |  |  |  |  |
| **FLU** |  | **g** | 0.23  0.8739 | 0.30  0.6954 | **0.85***  0.0839 | **0.91***  0.2435 |  | **g** | 0.12  0.9815 | 0.33  0.6302 | **1.06***  **0.0328** | **2.06***  **0.0015** |
|  |  | **p** |  |  |  |  |  | **p** |  |  |  |  |
| **DEXA** |  | **g** | 0.02  >0.9999 | − | **0.98***  0.0693 | − |  | **g** | 0.68  0.2885 | − | 0.40  0.6620 | − |
|  |  | **p** |  |  |  |  |  | **p** |  |  |  |  |

**Supplementary Table 3.** Inflammatory cytokine (TNF-α, IL-6, IL-1α, IL-1β, MCP-1) levels measured by quantitative RT-PCR in fluvoxamine- and dexamethasone-treated wild-type and *S1r^-/-^* mice. *g*>0.8 indicating large effect size calculated by Hedges’ g was considered significant as well as p<0.05 analyzed by one-way ANOVA followed by Dunnett’s multiple comparisons test and marked as bold, n=5-13 mice/group, *Hedges’g>0.8 vs corresponding group.

|  | **TNF-α** |  | **LPS** | | **PBS** | | **IL-6** |  | **LPS** | | **PBS** | |
| --- | --- | --- | --- | --- | --- | --- | --- | --- | --- | --- | --- | --- |
|  |  |  | **WT** | ***S1r*** ^-/-^ | **WT** | ***S1r*** ^-/-^ |  |  | **WT** | ***S1r*** ^-/-^ | **WT** | ***S1r*** ^-/-^ |
| **PBS** |  | **g** | **2.67***  **0.0001** | **0.89***  0.4273 | − | − |  | **g** | **2.27***  **<0.0001** | **1.99***  **0.0049** | − | − |
|  |  | **p** |  |  |  |  |  | **p** |  |  |  |  |
| **FLU** |  | **g** | **1.85***  **0.0054** | **0.80***  0.0712 | **2.22***  0.7006 | **1.33***  **0.0142** |  | **g** | **1.56***  **0.0020** | **1.46***  **0.0010** | **1.31***  0.6687 | **3.19***  **<0.0001** |
|  |  | **p** |  |  |  |  |  | **p** |  |  |  |  |
| **DEXA** |  | **g** | 0.05  0.9988 | − | **1.91***  **0.0009** | − |  | **g** | **1.00***  **0.0380** | − | **1.37***  0.1832 | − |
|  |  | **p** |  |  |  |  |  | **p** |  |  |  |  |
|  | **IL-1α** |  | **LPS** | | **PBS** | | **IL- 1β** |  | **LPS** | | **PBS** | |
|  |  |  | **WT** | ***S1r*** ^-/-^ | **WT** | ***S1r*** ^-/-^ |  |  | **WT** | ***S1r*** ^-/-^ | **WT** | ***S1r*** ^-/-^ |
| **PBS** |  | **g** | **2.67***  **<0.0001** | **0.92***  0.1956 | − | − |  | **g** | **2.32***  **<0.0001** | **1.11***  0.0797 | − | − |
|  |  | **p** |  |  |  |  |  | **p** |  |  |  |  |
| **FLU** |  | **g** | **2.03***  **<0.0001** | 0.49  0.3343 | **1.80***  0.7578 | **1.30***  **0.0284** |  | **g** | **1.67***  **0.0006** | 0.55  0.2244 | **2.40***  0.6380 | **1.67***  0.0040 |
|  |  | **p** |  |  |  |  |  | **p** |  |  |  |  |
| **DEXA** |  | **g** | **1.27***  **0.0060** | − | **1.56***  0.1240 | − |  | **g** | **1.09***  **0.0194** | − | **1.86***  0.1314 | − |
|  |  | **p** |  |  |  |  |  | **p** |  |  |  |  |
|  | **MCP-1** |  | **LPS** | | **PBS** | |  |  |  |  |  |  |
|  |  |  | **WT** | ***S1r*** ^-/-^ | **WT** | ***S1r*** ^-/-^ |  |  |  |  |  |  |
| **PBS** |  | **g** | **3.56***  **<0.0001** | **1.22***  0.1376 | − | − |  |  |  |  |  |  |
|  |  | **p** |  |  |  |  |  |  |  |  |  |  |
| **FLU** |  | **g** | **2.12***  **0.0009** | 0.44  0.4299 | **2.24***  0.2653 | **1.41***  **0.0270** |  |  |  |  |  |  |
|  |  | **p** |  |  |  |  |  |  |  |  |  |  |
| **DEXA** |  | **g** | **0.82***  0.1031 | − | **1.64***  **0.0080** | − |  |  |  |  |  |  |
|  |  | **p** |  |  |  |  |  |  |  |  |  |  |

**Supplementary Table 4.** Strain differences between respective treatments between wild-type and *S1r^-/-^* mice. Large effect size **(**Hedges’ g>0.8) was considered significant as well as p<0.05 analyzed by one-way ANOVA followed by Dunnett’s multiple comparisons test and marked as bold, n = 5-16 mice/group, *Hedges’g > 0.8 vs corresponding group.

|  |  | **WT PBS vs *S1r* ^-/-^ PBS** | **WT FLU vs *S1r* ^-/-^**  **FLU** |
| --- | --- | --- | --- |
| **Tidal volume** | **g** | **0.90***  0.2461 | 0.26  0.9737 |
|  | **p** |  |  |
| **Minute**  **ventilation** | **g** | **0.85***  0.1591 | 0.11  0.9996 |
|  | **p** |  |  |
| **Peak expiratory flow** | **g** | **0.95***  0.0769 | 0.04  0.9999 |
|  | **p** |  |  |
| **Mid-tidal expiratory flow** | **g** | 0.13  0.2356 | 0.33  >0.9999 |
|  | **p** |  |  |
| **Peak inspiratory flow** | **g** | 0.66  0.3338 | 0.59  0.6080 |
|  | **p** |  |  |
| **Frequency** | **g** | 0.14  0.9996 | 0.52  0.6926 |
|  | **p** |  |  |
| **Inspiratory time** | **g** | 0.14  0.9979 | 0.77  0.5488 |
|  | **p** |  |  |
| **Expiratory time** | **g** | 0.35  0.9312 | 0.39  0.9087 |
|  | **p** |  |  |
| **CD 68+ cells/ 10^6^ μm^2^** | **g** | 0.14  0.9996 | 0.58  0.8344 |
|  | **p** |  |  |
| **Neutrophil granulocytes** | **g** | 0.45  0.0146 | **1.08***  **0.0155** |
|  | **p** |  |  |
| **Lung index** | **g** | 0.12  0.9997 | **0.74***  0.3035 |
|  | **p** |  |  |
| **TNF-α** | **g** | 0.11  >0.9999 | **1.17***  **0.0059** |
|  | **p** |  |  |
| **IL-6** | **g** | **1.08***  0.9998 | **2.49***  **<0.0001** |
|  | **p** |  |  |
| **IL-1α** | **g** | **1.69***  >0.9999 | **1.13***  **0.0232** |
|  | **p** |  |  |
| **IL-1β** | **g** | 0.61  >0.9999 | **1.32***  **0.0112** |
|  | **p** |  |  |
| **MCP-1** | **g** | **2.15***  **0.9999** | **1.07***  **0.0256** |
|  | **p** |  |  |
| **Body weight** | **g** | **1.15***  0.2354 | **1.75***  **0.0009** |
|  | **p** |  |  |
| **Body weight change** | **g** | **0.83***  0.3648 | 0.15  0.9977 |
|  | **p** |  |  |
